# Supplementary figures and images for: Regional distribution of Christensenellaceae and its associations with metabolic syndrome based on a population-level analysis
Source: PeerJ. 2020 Aug 4;8:e9591. doi: 10.7717/peerj.9591 (PMC7413085; doi:10.7717/peerj.9591)

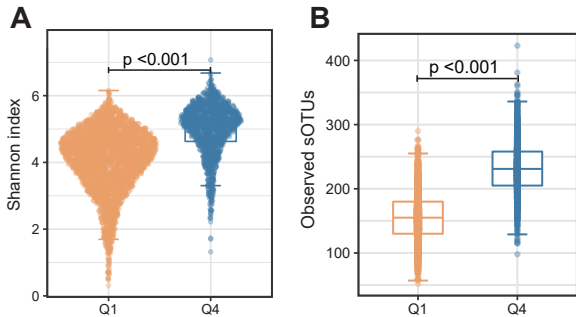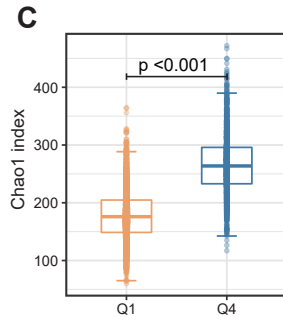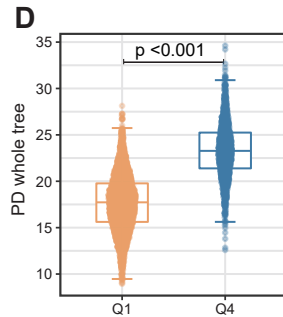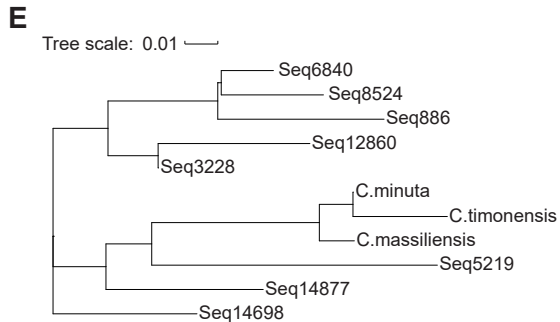

Supplement: Figure S1 — (A-D) Alpha-diversity index of the gut microbiota in two groups. Chao1, observed_OTUs, PD_whole tree and Shannon indices were compared between G1 (n=3316) and G2 (n=1465), p < 0.001. (E) Evolutionary relationships between 8 sequential taxonomic units with reading number more than 1000 and several reference genomes of 16S rRNA V4. [file peerj-08-9591-s001.pdf]

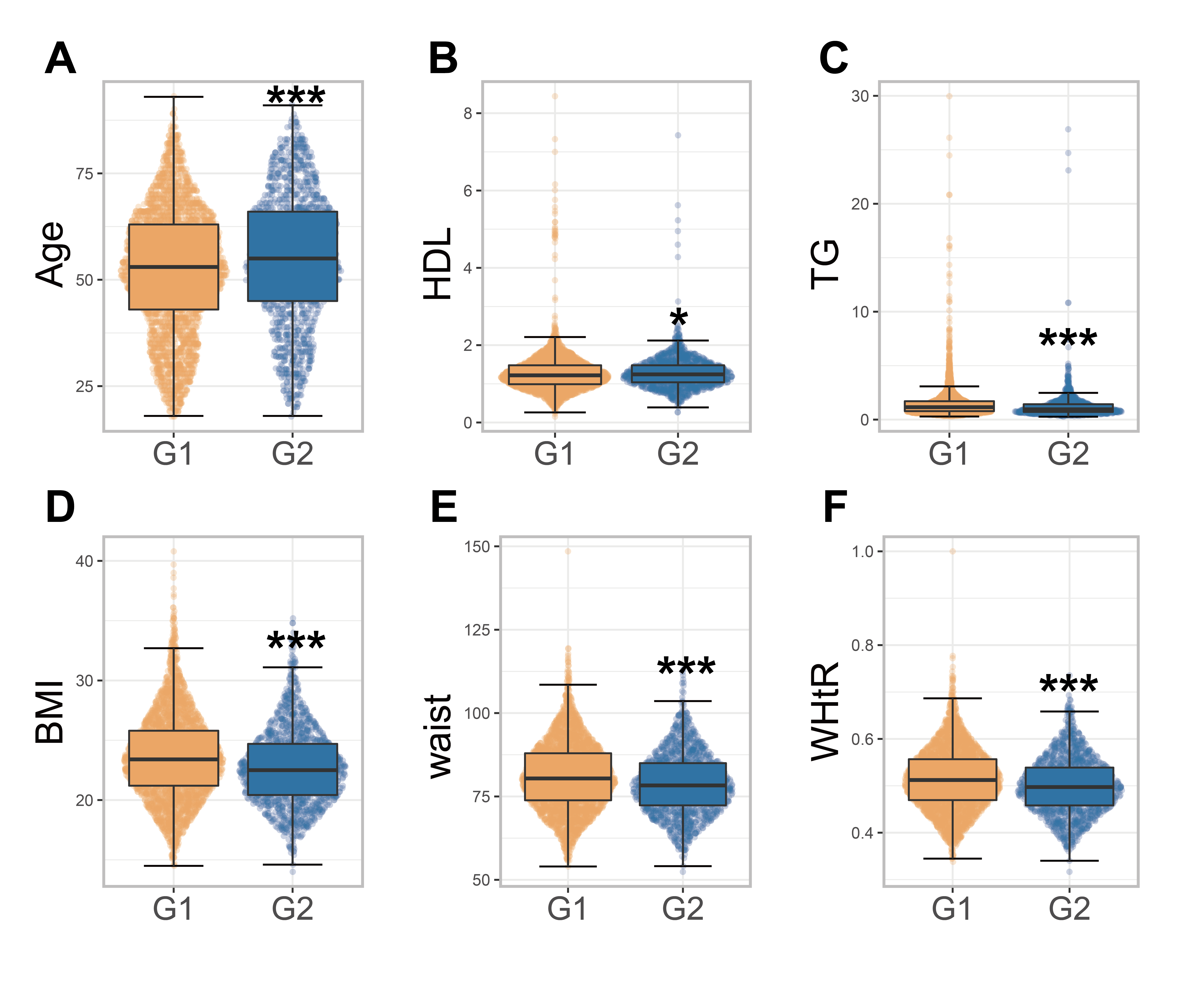

Supplement: Figure S2 — *** p < 0.001, ** p < 0.01, * p < 0.05 [file peerj-08-9591-s002.png]

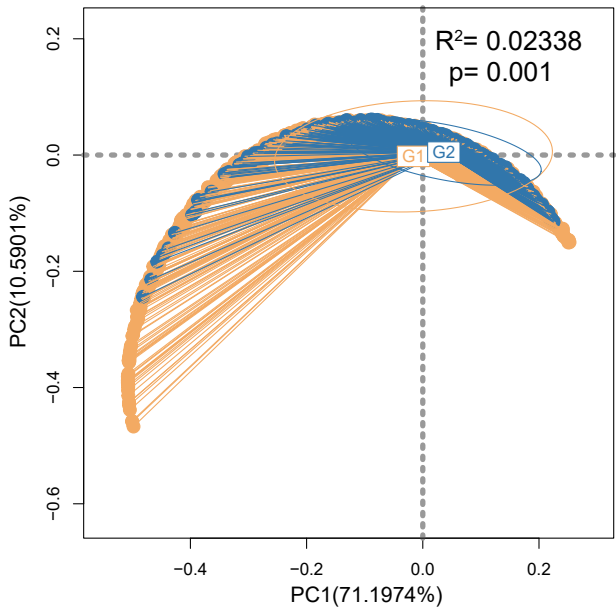

Supplement: Figure S3 [file peerj-08-9591-s003.pdf]
